# Relationship between the Asp1104His polymorphism of the nucleotide excision repair gene ERCC5 and treatment sensitivity to oxaliplatin in patients with advanced colorectal cancer in China

**DOI:** 10.6061/clinics/2017/e455

**Published:** 2018-11-23

**Authors:** Jiangying Kong, Zhuo Liu, Feng Cai, Xiaocheng Xu, Jun Liul

**Affiliations:** ^I^Clinical Laboratory Department, Zhejiang Xiaoshan Hospital, Hangzhou, Zhejiang 311202, China; II Department of Internal Medicine, Zhejiang Xiaoshan Hospital, Hangzhou, Zhejiang 311202, China; III Cancer Center of Zhejiang Xiaoshan Hospital, Hangzhou, Zhejiang 311202, China; IV Department of Oncology, The First People's Hospital of Xiaoshan, Hangzhou, Zhejiang 311201, China

**Keywords:** Polymorphism, ERCC5 Asp1104His, Advanced Colorectal Cancer, Treatment Sensitivity to Oxaliplatin

## Abstract

**OBJECTIVES:**

To study the relationship between the Asp1104His polymorphism of the nucleotide excision repair gene ERCC5 and treatment sensitivity to oxaliplatin in patients with advanced colorectal cancer (CRC) in China.

**METHODS:**

A group of 226 patients in the Department of Gastrointestinal Oncology at Zhejiang Xiaoshan Hospital from July 2011∼December 2016 and a control group of 226 normal healthy individuals were involved in this study. All patients were first diagnosed with advanced CRC and were treated with oxaliplatin-based chemotherapy. The genotype of ERCC5 at the site of amino acid 1104 was determined by a TaqMan probe-based real-time PCR approach.

**RESULTS:**

There were no differences in age or gender between the groups, but the percentages of smokers and individuals with a family history of cancer were significantly higher in the patient group than in the control group. Analysis of the G/C polymorphism frequency among the patients and the healthy controls showed that the frequencies of the CC genotype and the CC+GC genotype were significantly related to CRC, but no significant difference in these frequencies was found between genders. The analysis of the relationship between the 5-year survival rate and different genotypes showed that in the total patient group, regardless of gender, the 5-year survival rate was significantly associated with the Asp1104His polymorphism of ERCC5.

**CONCLUSIONS:**

The Asp1104His polymorphism of ERCC5 was associated with the risk and 5-year survival rate of CRC as well as treatment sensitivity to oxaliplatin.

## INTRODUCTION

Colorectal cancer (CRC) is a disease killing almost 60 thousand people every year 1. It is the third most common cancer in males, affecting approximately 10% of all male cancer patients, and is the second most common in females, affecting approximately 9.2% of all female cancer patients 2. Over 1.2 million new CRC cases have been diagnosed each year 3. CRC mainly affects the elderly, with a median age at diagnosis of 68 years, and CRC patients between 75 and 84 years old have the highest risk of cancer-related death 4.

To date, no clear causes of CRC carcinogenesis have been identified. However, genetic and environmental factors are generally thought to be associated with CRC development 5. Studies show that 20%-25% of CRC cases are associated with genetic and/or environmental risk factors related to CRC 6,7. Recently, the role of DNA damage and repair in CRC has attracted scholars' attention. It was reported that polymorphisms of some nucleotide excision repair (NER) genes, such as XPA(A23G), XPC(Lys939Gln), XPD(Lys751Gln, Asp312Asn), were related to CRC development 8. Studies also showed that mutations in genes involved in NER, including excision repair cross-complementation group 2 (ERCC2) Lys751Gln 9, excision repair cross-complementation group 1 (ERCC1) C118T 10 and excision repair cross-complementation group 5 (ERCC5) Asp1104His 11, were associated with CRC. However, to our best of our knowledge, there has been no research to date focusing on the relationship between treatment sensitivity to oxaliplatin, a key component of the standard first-line combination chemotherapy for metastatic CRC 12, and the Asp1104His polymorphism of ERCC5.

In the present study, we first investigated the relationship between the Asp1104His polymorphism of the nucleotide excision repair gene ERCC5 and treatment sensitivity to oxaliplatin in patients with advanced CRC in China. This study may provide more clinical evidence supporting roles for the ERCC5 Asp1104His polymorphism in treatment of CRC with oxaliplatin and may provide a deeper understanding of the occurrence and development of CRC.

## MATERIALS AND METHODS

### Patients

A total of 226 patients in the Department of Clinical Laboratory of Zhejiang Xiaoshan Hospital from July 2011∼December 2016 were involved in this study. The mean patient age was 59.23±6.54 years, and the male:female ratio was 144:82. All patients were first diagnosed with advanced CRC (stage III or IV according to the American Joint Committee on Cancer (AJCC) cancer stage classification 13 by histopathology using colonoscopy. Additionally, a control group of 226 normal healthy individuals, with a mean age of 58.13±7.29 years and a male:female ratio of 132:94, was recruited. All individuals in the control group were confirmed to not have a history of any type of cancer and to not have digestive system disease during the study period.

Basic clinical characteristics such as weight, height, smoking habits, alcohol use and personal/family medical history were collected for all participants in the study using a questionnaire. All patients were treated with chemotherapy based on oxaliplatin 14. Briefly, a modified FOLFOX4 regimen was adopted as follows: oxaliplatin 130 mg/m^2^, *iv* in 3h on day l; calcium folinate (CF) 130 mg/m^2^, *iv* in 2h on days 1∼5; fluorouracil (5-FU) 300 mg, *iv* in 4h on days 1∼5; repeated in 3 weeks. Only patients who completed at least 2 cycles of chemotherapy were recruited into our study. Responses to treatment were defined by 4 categories according to the Response Evaluation Criteria in Solid Tumors (RECIST) 15: complete response (CR), partial response (PR), stable disease (SD) and progressive disease (PD). Response was defined as a decrease of at least 50% in the initial tumor size. All patients were further divided into groups of responders, which included patients categorized as CR or PR, and nonresponders, which included patients categorized as SD or PD. Informed consent was obtained from all participants in the study. This study was approved by the Ethics Committee of Zhejiang Xiaoshan Hospital.

### Genotyping

For each participant, 4 ml of anticoagulated peripheral blood sample was collected and stored at -20°C before the study. DNA was extracted according to the manufacturer's instructions using the Qiagen Blood DNA Mini Kit (Qiagen Inc., Valencia, CA). The genotypes of the ERCC5 Asp1104His polymorphism were determined by a TaqMan probe-based real-time polymerase chain reaction (PCR) approach. Every reaction contained 20 ng of genomic DNA, 0.4 µL of the primers and probe mixture (single-nucleotide polymorphism TaqMan assay mix), 7.5 µL of universal PCR mixture (TaqMan genotyping master mix) and water to 15 µL. The primers used in the study were as follows: Asp1104His, forward 5'-GACCTGCCTCTCAGAATCATC-3', reverse 5' CCTCGCACGTCTTAGTTTCC-3'. Briefly, DNA was first denatured at 94°C for 10 min to activate the Taq polymerase, followed by initial denaturation with 45 cycles at 94°C for 1 min, 60°C for 1 min and a final extension at 72°C for 5 min. Ten percent of the subjects were randomly selected for repeat analysis, and the evaluation was considered to be finalized when 100% concordance was observed for the repeat test results compared with the original test results. Plates were read by an ABI 7900 real-time PCR instrument (Applied Biosystems, Nærum, Denmark). The genotyping results were analyzed by ABI SDS2.3 software.

### Statistical Analysis

Polymorphic genotypes were categorized into homozygous wild type, heterozygous and homozygous variant. Continuous and categorical variables were expressed as the mean ± SD and n (%) of study participants, respectively. Independent continuous variables were compared using Student's t-test or ANOVA, and categorical data were compared using the chi square test or Fisher's exact test to assess Hardy-Weinberg equilibrium. For survival analysis and evaluation of differences in the 5-year survival rate, Kaplan-Meier analysis and the log-rank test were used. Follow-up started on the date of completion of chemotherapy and extended to the date of death or the last follow-up visit. Follow-up lasted for the first 5 years followed by once a year thereafter. Logistic regression analysis was used to adjust for age, sex, and smoking and family history of cancer. Comparison of cross-products [odds ratio (OR)] with a 95% confidence interval (95% CI) was also recorded. A *p*-value of less than 0.05 was considered statistically significant. All analyses were conducted using SPSS 18.0.

## RESULTS

### Demographic and Basic Clinical Characteristics of the Patients

The demographic and basic clinical characteristics of all participants are shown in Table 1. A total of 226 patients, including 144 males and 82 females, were involved in this study; the mean patient age was 59.23±6.54 years. A control group of 226 normal healthy individuals, including 132 males and 94 females, with a mean age of 58.13±7.29 years was also recruited. No significant differences in age, gender, or body mass index (BMI) were observed between the two groups. However, the percentages of smokers and individuals with a family history of cancer were significantly higher in the patient group than in the control group, *p*<0.05. Among all patients, 126 (56.0%) were diagnosed with stage III disease, and 100 (44.0%) were diagnosed with stage IV disease. Additionally, 144 (63.7%) patients were sensitive to oxaliplatin-based chemotherapy, with 74 cases of CR and 70 cases of PR, and 82 (36.3%) patients were nonsensitive to oxaliplatin-based chemotherapy, with 46 cases of SD and 36 cases of PD.

### Association of the Asp1104His Polymorphism of ERCC5 with the Risk of CRC

As shown in Table 2, the frequency of the G/C polymorphism was compared between patients and healthy controls as well as between male and female patients. The results showed that the frequencies of the CC genotype and CC+GC genotype were significantly related to CRC, *p*<0.05. No significant difference was found between different genders.

**Figure 1:**
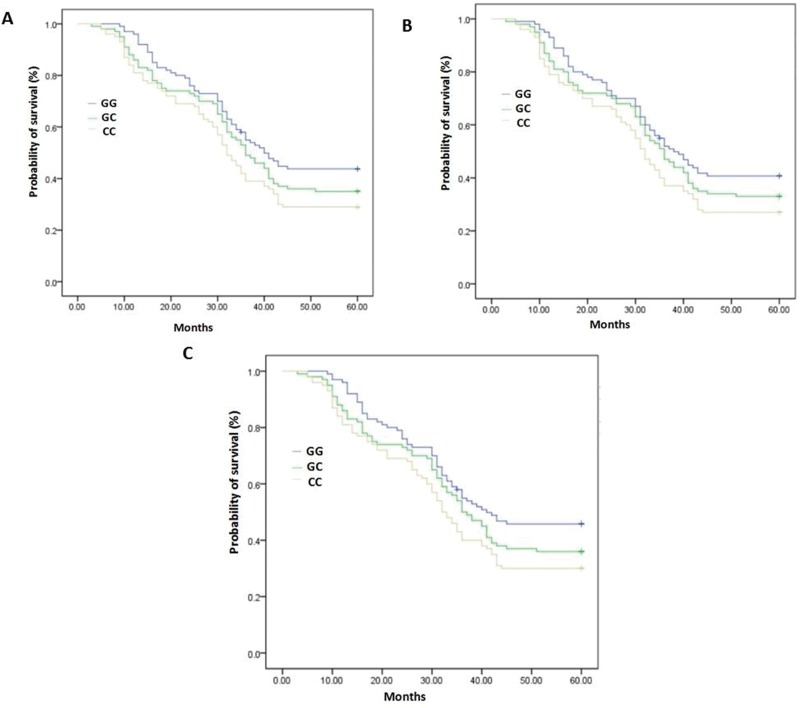
A. Kaplan–Meier survival estimation for all patients. B. Kaplan–Meier survival estimation for male patients. C. Kaplan–Meier survival estimation for female patients.

A log-rank test was used to evaluate the association of the 5-year survival rate with the ERCC5 Asp1104His polymorphism status. In the total patient group, the 5-year survival rate was significantly associated with the Asp1104His polymorphism of ERCC5, *p*<0.01. Moreover, in both male and female patients, the 5-year survival rate was significantly associated with the Asp1104His polymorphism of ERCC5, *p*<0.05 (Table 3, Figure 1). Among all genotype groups, the survival rate was the lowest in the CC group.

### Association of the Asp1104His Polymorphism of ERCC5 with Treatment Sensitivity to Oxaliplatin

Lastly, the association between the Asp1104His polymorphism of ERCC5 and treatment sensitivity to oxaliplatin was analyzed, and the results are shown in Table 4. In all patients, the frequencies of GC, CC and GC+CC were significantly associated with treatment sensitivity to oxaliplatin, *p*<0.05. The results for male patients were similar to those for all patients; however, in female patients, only the GC and GC+CC genotypes showed significant associations with the treatment response frequency.

## DISCUSSION

Although there are numerous studies on CRC, the exact mechanisms for its occurrence and development are still unknown. Genetic factors are considered key components in the development of CRC. One study showed that family history of cancer is one of the strongest predictors of CRC risk and that this risk increases with an increasing number of affected relatives, especially when CRC occurs at a young age 2. Recently, the relationship between CRC and polymorphisms of NER genes has been frequently reported. Studies focusing on ERCC2 Lys751Gln 16 and ERCC1 C118T 17 showed that these two genes might play key roles in CRC. Some studies also demonstrated that the ERCC2 Lys751Gln and ERCC1 C118T mutations were associated with treatment sensitivity to oxaliplatin 18. However, to the best of our knowledge, no research to date has focused on the relationship between treatment sensitivity to oxaliplatin and the Asp1104His polymorphism of ERCC5.

In the present study, we first investigated the relationship between the Asp1104His polymorphism of the nucleotide excision repair gene ERCC5 and treatment sensitivity to oxaliplatin in patients with advanced CRC in China. A group of 226 CRC patients and a control group of 226 normal healthy individuals were involved in this study. There were no differences in age or gender between the study groups, but the percentages of smokers and individuals with a family history of cancer were significantly higher in the patient group than in the control group.

Next, we analyzed the frequency of the G/C polymorphism among the patients and the healthy controls. The results showed that the frequencies of the CC and CC+GC genotypes were significantly related to CRC. No significant differences between genders were found. Du et al. 19 analyzed the association between the ERCC5 Asp1104His polymorphism and CRC risk in a Chinese population and found that C genotype was significantly related to CRC risk, which was consistent with the results of our study. In the total patient group, regardless of gender, the 5-year survival rate was significantly associated with the ERCC5 Asp1104His polymorphism status. Schrama et al. 20 studied the relationship between ERCC5 Asp1104His and melanoma and found that this polymorphism was significantly associated with the 5-year survival rate, which was similar to the findings of our study. In addition to its association with CRC, the ERCC5 Asp1104His polymorphism was considered to be related to HIV 21 and various cancers, such as breast and lung cancer 22, and it was associated with tumor stage and grade 23.

Finally, the results of the association of the ERCC5 Asp1104His polymorphism with treatment sensitivity to oxaliplatin showed that the frequencies of GC, CC and GC+CC were significantly associated with treatment sensitivity to oxaliplatin in the total patient group. The results for male patients were similar to those for all patients. However, in female patients, only the GC and GC+CC genotypes showed significant associations with the treatment response frequency.

In conclusion, we first investigated the relationship between the Asp1104His polymorphism of the nucleotide excision repair gene ERCC5 and treatment sensitivity to oxaliplatin in patients with advanced CRC in China. The results showed that the ERCC5 Asp1104His polymorphism was associated with the risk and 5-year survival rate of CRC as well as treatment sensitivity to oxaliplatin. This study may provide more clinical evidence supporting roles for ERCC5 Asp1104His in treatment of CRC with oxaliplatin and may provide a deeper understanding of the occurrence and development of CRC.

## AUTHOR CONTRIBUTIONS

Kong J wrote the manuscript. Liu Z and Cai F contributed to the discussion. Xu X and Liu J contributed to the discussion and commented on an earlier version of the manuscript. All authors read and approved the final version of the manuscript.

## Figures and Tables

**Table 1:** Demographic and basic clinical characteristics of the patient and control groups.

Characteristics	Patients, n=226	Controls, n=226	*p-*value
Mean age, years	59.23±6.54	58.13±7.29	0.45
Gender, male:female	144:82	132:94	0.38
BMI, kg/m^2^	25.47±3.59	26.31±5.15	0.86
Smoker, n (%)	160 (70.8)	82 (36.3)	<0.001
Family history of cancer, n (%)	24 (10.2)	2 (0.9)	<0.001
Tumor stage			
III	126 (56.0)		
IV	100 (44.0)		
Oxaliplatin treatment response			
Responder (%)	144 (63.7)		
CR	74		
PR	70		
Nonresponder (%)	82 (36.3)		
SD	46		
PD	36		

BMI: body mass index, CR: complete response, PR: partial response, SD: stable disease, PD: progressive disease.

**Table 2:** Association of the ERCC5 Asp1104His polymorphism with the risk of CRC.

	Controls, (%)	Patients, (%)	Adjusted* OR (95% CI)	*p*-value	Male patients, (%)	Female patients, (%)	Adjusted* OR (95% CI)	*p-*value
GG	95 (42)	70 (31)	1.00 (reference)	–	44 (30)	26 (32)	1.00 (reference)	–
GC	83 (37)	112 (50)	1.57 (0.82-1.55)	0.01	70 (49)	42 (51)	1.35 (0.93-1.65)	0.46
CC	48 (21)	44 (19)	1.31 (0.93-1.69)	0.75	30 (21)	14 (17)	1.42 (0.86-1.74)	0.13
GC+CC	131 (58)	156 (69)	1.29 (0.96-1.87)	0.03	100 (70)	56 (68)	1.27 (0.87-1.53)	0.61

* Adjusted for age, sex, and smoking and family history of cancer in the logistic regression model

**Table 3:** Relationship between 5-year survival rate and ERCC5 Asp1104His polymorphism status.

	All patients, 5-year survival rate (95% CI)	*p-*value	Males, 5-year survival rate (95% CI)	*p-*value	Females, 5-year survival rate (95% CI)	*p-*value
GG	0.43 (34-45)	0.011	0.40 (32-43)	0.017	0.45 (32-39)	0.010
GC	0.35 (32-38)		0.33 (32-39)		0.36 (29-34)
CC	0.29 (30-33)		0.27 (30-33)		0.30 (32-37)

**Table 4:** Association of the Asp1104His polymorphism of ERCC5 with treatment sensitivity to oxaliplatin.

	Responders, n (%) n=144	Nonresponders, n (%) n=82	Adjusted* OR (95% CI)	*p-*value
All patients				
GG	53 (37)	17 (30)	1.00 (reference)	-
GC	66 (46)	46 (57)	1.34 (0.85-1.55)	0.03
CC	25 (17)	19 (23)	1.37 (0.79-1.89)	0.04
GC+CC 285	91 (63)	65 (80)	1.41 (0.97-1.76)	0.01
Males	n=92	n=52		
GG	35 (38)	9 (17)	1.00 (reference)	-
GC	41 (45)	29 (56)	1.26 (0.94-1.67)	0.04
CC	16 (17)	14 (27)	1.45 (0.83-1.86)	0.04
GC+CC 184	57 (62)	43 (83)	1.51 (1.03-2.19)	0.01
Females	n=52	n=30		
GG	22 (36)	8 (30)	1.00 (reference)	-
GC	25 (48)	17 (55)	1.28 (1.01-1.73)	0.04
CC	9 (16)	5 (15)	1.36 (0.95-1.77)	0.23
GC+CC	34 (64)	22 (70)	1.45 (1.13-1.95)	0.04
